# Impact of malnutrition on the outcome and length of hospital stay in elective pediatric surgical patients: prospective cohort study at tertiary hospitals in Ethiopia

**DOI:** 10.1186/s40795-023-00788-9

**Published:** 2023-11-09

**Authors:** Belachew D. Wondemagegnehu, Woubedel K. Aklilu, Milliard D. Beyene, Bareng A. Sanny Nonyane

**Affiliations:** 1 College of Health Sciences, Department of Surgery, Addis Ababa University, 1176 Churchill Rd, Zambia St, Lideta sub-city, Addis Ababa Ethiopia; 2 Department of International Health, Johns Hopkins Bloomberg School of Public Health, Baltimore, MD US

**Keywords:** Malnutrition, Nutrition, Pediatric Surgery, Surgical outcome

## Abstract

**Background:**

Pediatric surgical patients in low and middle-income countries suffer from malnutrition on top of the surgical pathology and post-operative stress which increases post-operative morbidity and mortality. Malnutrition is highly prevalent in Africa and is expected to impact the outcome of surgical patients. The study was aimed at determining the impact of malnutrition on the outcome and length of hospital stay in elective pediatric surgical patients.

**Methods:**

A prospective study was done on children, aged one month to fourteen years, who had undergone elective general surgery. Upon admission, nutritional assessment using an anthropometric indicator was undertaken and used to derive the WHO Anthro Z scores. Malnutrition was defined as a binary outcome: severely/moderately malnourished (Z < -2) versus well nourished (Z ≥ -2). After surgery, postoperative complications were documented. Chi-squared tests and t-tests were used to assess associations.

**Results:**

Of the 109 enrolled children, 49 (45%) had malnutrition preoperatively. Infants had a higher prevalence of malnutrition (65% versus 35%, p-value = 0·028) compared to older children. Postoperative infection was relatively more common in malnourished children (27·1% versus 20%). The mean post-operative stays were 5·69 days (SD 0.46) for well-nourished children and 6.89 days (SD 0·9) for malnourished patients but the difference was not statistically significant.

**Conclusion:**

We observed neither significant long hospital stays nor a higher incidence of postoperative infection among children with malnutrition. Further investigations with a larger sample size are warranted.

**Supplementary Information:**

The online version contains supplementary material available at 10.1186/s40795-023-00788-9.

## Introduction

Adequate nutrition is essential in early childhood to ensure healthy growth, proper organ formation and function, a strong immune system, and neurological and cognitive development [1, 2]. Malnutrition is a deficiency state of both macro and micronutrients and their overconsumption, causing measurable adverse effects on human body structure and function resulting in specific physical and clinical outcomes. Worldwide, it is estimated that more than one-third of under-five deaths are attributable to undernutrition [1, 3].

In Sub-Saharan African countries, suffering from poverty as well as drought, children are highly affected by malnutrition. It is one of the leading causes of morbidity and mortality in children under five years of age [4]. According to a 2016 report from Ethiopia Demographic and Health Survey, the prevalence of stunting and being underweight in children under 5 years is 38% and 24% respectively, indicating that it is the most serious public health problem in Ethiopia [1, 5].

On the other hand, surgery is a traumatic condition that can lead to the depletion of body stores due to a catabolic state and decreased immunocompetence [2, 6]. Based on studies done in Canada, Japan, and Brazil malnourished children undergoing surgery have been shown to have an increased risk of complications following surgery. These include infections, increased length of hospital stays, prolonged postoperative assisted mechanical ventilation, and increased mortality [6–9]. The reason is, malnutrition reduces the body’s immunity and stress resistance on top of surgical trauma [10].

A high prevalence of malnutrition, up to 40% has been shown in pediatric surgical patients in various clinical reviews [11, 12]. This increased prevalence is seen especially with pathologies associated with gastrointestinal (GI) anomalies where most of our patients are affected [13–15]. Malnutrition in these patients can be the result of several factors: malabsorption due to limited function of the GI tract, poor utilization of nutrients due to altered function of the GI system, decreased oral intake, and/or tumor-related cachexia. Thus, it is recommended to put them on enteral or parenteral feeding during the perioperative period to maintain and possibly improve their nutrition status before undergoing surgery [2, 13].

The burden of malnutrition among hospital-admitted pediatric medical patients has been studied in Ethiopia [16]. But there is no similar data concerning pediatric surgical patients’ nutritional status and malnutrition effect on postoperative outcome. Therefore, we conducted a prospective study to evaluate the preoperative prevalence of malnutrition and its impact on postoperative outcomes.

## Methods

### Study design and setting

A prospective cohort study was conducted on elective pediatric surgical patients operated in two governmental pediatric surgical full-time centers, Menelik II specialize hospital (MSH) (from Oct 1 to 31, 2019) and Tikur Anbessa specialized hospital (TASH) (from Jan 1, 2020, to March 30, 2020) in Addis Ababa, Ethiopia. The two-month gap was because of renovation issues and patient distribution.

### Study population

All elective pediatric surgical patients from age 29 days to 14 years who were operated on during the study period and did not undergo any other surgery in the last 30 days, includes: Gastrointestinal, Urologic, Thoracic, and Neck surgeries.

#### Exclusion criteria

Those admitted for less than 24 h, those admitted for endoscopic procedures, Surgical Oncologic patients, and other Pediatric surgeries like neurosurgery, Ear Nose Throat(ENT), and orthopedic surgeries.

### Data management

Upon admission, nutritional histories were taken, and after anthropometric measurements were done structured interviewer-administered questionnaires were filled. Post operative complications were defined as presence of complications not related to the direct surgical intervention, and the length of hospital stay more than expected for a certain procedure was taken as prolonged hospital stay. Since there is no consensus on which nutritional assessment tool is standard in predicting postoperative outcomes, all methods of evaluation were included **(Annex-1)**. An average of duplicated measurements of weight, height, and mid-upper arm circumference was taken for each study participant. Laboratory investigations focusing on acute and chronic malnutrition status, albumin, and prealbumin were sent before the operation for only the first 67 patients because of shortage of lab reagents. After surgery, the patients were monitored for post-operative complications, up to 30 days of post-operation either in person or through a telephone interview. Malnutrition was defined by anthropometric measures from which we derived the WHO Anthro Z-scores (*Weight-for-Height, Weight-for-age, Height-for-Age, Body Mass Index, and Mid-upper arm circumference*) were derived. Patients were categorized as severely/moderately malnourished (Z < -2) or well nourished (≥ -2) for any of the Z scores. For Down syndrome patients, we used an anthropometric definition designed for them.

### Statistical analysis

Data were entered into Microsoft Excel and cleaned; analysis was done using SPSS version 25. Descriptive statistics were computed for independent and dependent variables. Chi-square and Fisher’s exact tests - were used to evaluate the association between the binary outcomes (prevalence of malnutrition at admission and having post-operative complications) and independent variables (age, sex, maternal education status, Wealth index, and diagnosis). Non-parametric t-tests were used to evaluate the association between the length of hospital stay and the independent variables. Multivariate regression analyses were not done as there was no significant association found in bivariate analyses for more than one independent variable. Associations with a p-value < 0·05 were defined as statistically significant.

## Result

A total of 109 patients were recruited during the study period comprising 90 (82.6%) male and 19 (17·4%) female patients. The mean age was 55·6 months SD ± 47·1 with a range of 1 month–14 years. One-third of the patients came from the main city, Addis Ababa. The education level of the mothers was assessed based on school level, and 52·2% (51/97) had attended elementary school whereas 15·5% (15/97) had no schooling. The wealth index was calculated using the Ethiopia DHS-2016 equity tool and 94·5% (103/109) were found to be living in adequate conditions per national standards (Table 1).

**Table 1:** Sociodemographic characteristics and diagnosis of the study participants (n = 109) on admission

Characteristics	Frequency	Percent
**Age (month)**		
*≤ 12*	23	21·1
*13–60*	51	46·8
*> 60*	35	32.1
**Sex**		
*Male*	90	82·6
*Female*	19	17.4
**Maternal education level**		
*≤ 8*	51	46·8
*9–12*	16	14·6
*> 12*	15	13·8
*None*	15	13·8
*Missing*	12	11·0
**Residence**		
*Urban*	78(71.6)	71·6
*Rural*	28(25.7)	25·7
*Missing*	3(2.7)	2·7
**Wealth Index**		
*Adequate*	103 (94.5)	94·5
*Relatively poor*	6 (5.5)	5·5
**Diagnosis**		
urology	42(38.5)	38·5
GI	49(45.0)	45·0
Chest	2(1.8)	1·8
Inguinal hernia	11(10.1)	10·1
Others	5(4.6)	4·6

*Sociodemographic Characteristics and Diagnosis of 109 Children Admitted and operated on elective bases and assessed for their nutritional status and its impact on their postoperative course at Tikur Anbessa and Menellek II tertiary Hospitals, Ethiopia from Oct.2019 to Mar.2020*

The majority of the patients, 49 (45%), presented with gastrointestinal-related problems like Anorectal malformation and Hirschsprung disease; urologic disease accounted for 38.5% (42/109) including hypospadias. The rest of the patients presented with inguinal, chest problems.

and benign masses (in the abdomen, sacrum, and neck) 10.1%, 1.8%, and 4.6% respectively. 9.2% (10/109) of them have comorbidities of which four of the patients have Down Syndrome, 2.

cardiac anomalies, two GI anomalies, one VACTERAL, HIV, and hypothyroidism. One-fourth of patients [23.9% (22/92)] have hemoglobin levels below 10 g/dl (Table 2).

Infants (aged 1 month to 1 year) had a higher prevalence of malnutrition than other age groups (65% versus 35%, p = 0.028). In terms of wasting, specifically, infants had a higher prevalence as well (39.1% 9/23, p-value = 0.008). More females were affected by malnutrition (63.2% Vs 41.1%, p = 0.079) than male patients even though it is not statically significant. The mother’s educational status and calculated wealth index were not associated with nutritional status. It is expected to have poor nutritional status in patients with gastrointestinal disease conditions. We found that patients with GI disease had a relatively higher prevalence of malnutrition than those with other diseases (55.1% vs. 36.7%, p = 0.054), however, was not significant.

Among 108 patients, 13.9% (n = 15) developed postoperative infection and 12% (n = 13) developed other complications. One patient was not included in the association analysis as the operation was canceled. 20.8% of the children with malnutrition had a post-operative infection while 8.3% of those without malnutrition had an infection (P = 0.06). More than half of the infections were surgical site infections. Children with malnutrition developed more complications in general than well-nourished children (27.1% Vs 20%, p = 0.39). There was one in-hospital mortality secondary to chest focus sepsis.

**Table 2:** Bivariate analysis of the nutritional status and determining factors

	Well-nourished n (%)	Malnourished n (%)	P value
**Age (month)**			0.028*
*≤ 12*	8(34.8)	15(65.2)	
*> 12*	52(60.5)	34(39.5)	
**Sex**			0.079
*Male*	53(58.9)	37(41.1)	
*Female*	7(36.8)	12(63.2)	
**Diagnosis**			0.177^1^
*Urology*	26(61.9)	16(38.1)	
*GI*	22(44.9)	27(55.1)	
*Chest*	2(100)	0(0)	
*Inguinal*	8(72.7)	3(27.3)	
*Others*	2(40.0)	3(60.0)	
**Hemoglobin**			0.400
*<10 g/dl*	10(45.5)	12(54.5)	
*≥10 g/dl*	39(55.7	31(44.3	
**Subjective assessment**			0.100
*Well-nourished*	56(28.3)	40(41.7)	
*Moderate/severely malnourished*	4(33.3)	8(66.7)	
**Albumin**			1.00^1^
< 3.5 g/dl	2(66.7)	1(33.3)	
≥3.5 g/dl	44(67.7)	21(32.3)	
**Prealbumin**			0.437
*<0.17 g/l*	20(62.5)	12(37.5)	
*≥0.17 g/l*	25(71.4)	10(28.6)	
**Infectious complication**			0.062
Yes	5(33.3)	10(66.7)	
No	55(59.1)	38(40.9)	
**Noninfectious complication**			0.643
Yes	8(61.5)	5(38.5)	
No	52(54.7)	43(45.3)	

^*1*^ *Exact fisher test done for values < 5*

*Bivariate analysis of the nutritional status and determining factors of 109 Children admitted and operated on elective bases at Tikur Anbessa and Menellek II tertiary Hospitals, Ethiopia from Oct.2019 to Mar.2020*

The mean postoperative hospital stays for well-nourished and malnourished children were 5.69 days (SD 0.46) and 6.89 days (SD 0.9), respectively. The mean difference was assessed by a t-test and it was not statistically significant, p = 0.77. Associations with independent variables: albumin, prealbumin, hemoglobin, and diagnosis type were assessed. However, none of these independent variables were associated with postoperative complications, mainly referring to hospital acquired infections like wound infections, pneumonia, UTI, and wound failures., or length of hospital stay (Table 3).

**Table 3:** Bivariate and nonparametric analysis of outcome measurement in postoperative children after elective surgery

	Post-operative complication		Length of hospital stay^1^	
	n (%)	*p-value*	Mean ± SD	*p- Value*
**Age (month)**		*0·607*		*0·864*
*≤ 12*	6(27·3)		7·8(1.52)	
*> 12*	19(22·1)		5·8(0.46)	
**Sex** ^**2**^		*0·767*		*0·740*
*Male*	20(22·5)		6·0(0·51)	
*Female*	5(26·3)		7·3(1·31)	
**Diagnosis**		*0·959*		*0·150*
*GI*	11(22·9)		7·5(0.86)	
*others*	14(23·3)		5·2(0.46)	
**Hemoglobin**		*0·697*		*0·396*
*< 10 g/dl*	6(27·3)		8·2(1·56)	
*≥ 10 g/dl*	16(23·2)		5·62(0·52)	
**Subjective assessment** ^**2**^		*0·461*		*0·938*
*well*	20(21·1)		5·96(0·46)	
*Moderate/sever*	4 (33·3)		7·9(2·4)	
**Prealbumin**		*0·948*		*0·211*
*< 0.17 g/l*	6(19·4)		6·7(0·94)	
*≥ 0.17 g/l*	7(20·0)		4·8(0·63)	
**Albumin** ^**2**^		*0·377*		*0·94*
< 3.5 g/dl	1(50·0)		11(14·14)	
≥ 3.5 g/dl	13(20·0)		5.5(3·61)	
**Nutritional Status**		*0·386*		*0·770*
*Well-nourished*	12(20·0)		5·7(0·46)	
*Malnourished*	13(27·1)		6·9(0·9)	

^*1*^ *P values determined by Mann-Whitney nonparametric test.* ^*2*^ *Exact fisher tests were done for values < 5 in chi-square analysis*

*Bivariate and nonparametric analysis of outcome measurement and impact of their nutritional status on postoperative course in 109 children after elective surgery at Tikur Anbessa and Menellek II tertiary Hospitals, Ethiopia from Oct.2019 to Mar.2020*

## Discussion

Malnutrition remains one of the main health problems prevalent among Ethiopian children [5]. This was also observed in our pediatric surgical patients with a prevalence rate of 45%. To our knowledge, there is no other similar study in the country to compare our results. Although the observed prevalence of wasting, stunted, and underweight was lower in comparison to the Ethiopian demographic and health survey, a comparable estimate of malnutrition was seen in community-based studies done in a rural part of Ethiopia,48.5% by Endris N et al. [17].

Studies done on pediatric surgical patients in other low- and middle-income countries have revealed high prevalence rates of malnutrition. Adigun and Ogun Doyin from Nigeria reported a 46.2% prevalence, and from India, Pooja and Dave et al. reported a 46% prevalence [12, 18]. These results are higher than the reported prevalence from higher-income countries like Germany (6·1%), France (11%), and Brazil (6·9%) [11]. The difference could be explained by good economic status with good healthcare systems in high-income countries.

Infants were more likely to be malnourished than older children similar to reports by Barutçu A. and Barutçu S [19]. Similarly, the Nigerian study showed that children aged 1 year and below are 4·28 times more likely to suffer from malnutrition than older ones [12]. Likewise, in the study done by Ross et al., on the cardiac surgical patient the infants were found to have higher rates of malnutrition than neonates and older children [20]. On the contrary, from community-based studies of prevalence as the age of a child increases the risk of being malnourished increases [17].The difference between the community report and our result could be because 78.3% of the infants were having gastrointestinal anomalies and disease which result in poor intake and malabsorption affecting their nutritional status.

Factors that could affect the prevalence of malnutrition in other studies like maternal education level, and the economic status of the family didn’t show any association in our study [17, 21]. This could be because 73·6% of the patients came from urban settings, and were a homogeneous group in terms of the wealth index. even though more female patients were malnourished compared with male patients, their number is small to conclude.

On the assessment of the impact of malnutrition on postoperative complications, there are controversial results with clear associations in some and no correlation in others. Two systemic reviews were done to answer and clarify the effect of nutritional status on postoperative outcomes. Wessner et al., were trying to determine if nutritional assessment impact clinical outcome [7]. They found weak evidence of preoperative nutritional assessment being predictive of adverse clinical outcomes in pediatric surgical patients. However, this review only identified six studies in total, five of which were undertaken in a pediatric cardiac surgical patient, limiting the validity of these findings to other non-cardiac surgical patients. The other review was done by R. Hill et al., on the prognostic effect of undernutrition on infectious complications in children undergoing surgery. They analyzed twelve studies of which only four of them deal with general surgical cases. Even though there was some evidence showing a relationship between undernutrition and the risk of developing any infection-related complication again the evidence was weak [22]. One of the challenges faced by both reviews was the uniformity of nutritional assessment as well as the heterogeneity of the disease conditions. Hence, they failed to conclude.

In our study, even if infectious complications were seen more in malnourished patients, it was not statistically significant (p = 0·06). This result is consistent with the study done in Nigeria [12]. However, Secker et al. reported a significantly higher postoperative infection rate in the malnourished population versus the well-nourished group (p = 0·02) [8]. They used the Subjective Global Nutritional Assessment tool to determine nutritional status. But when they use the objective measures, no association was observed except height-for-age and postoperative length of stay. With the high prevalence of malnutrition in our patients and the immunosuppressive effect on surgical patients, it would be expected to have a high rate of infection. Nonetheless, the lower sample size in our study may be the reason for the non-significant findings.

Assessing the effect on the length of hospital stay was important not only to estimate the hospital cost and economic impact but also to determine the turnover rate of our patients because of the long waiting list. However malnourished patients did not have significantly longer hospital stay compared with well-nourished patients. And also, the biochemical profiles and the subjective assessment method failed to predict the effect on the length of postoperative stay. The limitations of the study were, the biochemical profile was not assessed for all patients, the lack of a validated subjective nutritional assessment tool suitable for our setup, and heterogeneity of the disease may affect the outcome measurement as some disease conditions may need longer hospital stay without any complications.

## Conclusion

In this study, we found that poor nutritional status was not significantly associated with postoperative outcomes. However, based on our observation and other studies congenital gastrointestinal anomaly and younger age (infants) were identified to be more exposed to poor nutrition. Evaluating the nutritional status of surgical patients should be part of routine physical examination as there’s evidence of an association with the outcome of surgery in other studies. Thus, future studies with larger sample sizes should focus on this group of patients. As malnutrition is a modifiable risk factor, morbidity and mortality following could be improved by perioperative rehabilitation.

### Electronic supplementary material

Below is the link to the electronic supplementary material.

Supplementary Material 1

## Data Availability

The data set used and or analyzed during the current study area are available from the corresponding author upon a reasonable request.
