# Supplementary material for: Impact of malnutrition on the outcome and length of hospital stay in elective pediatric surgical patients: prospective cohort study at tertiary hospitals in Ethiopia
Source: BMC Nutr. 2023 Nov 9;9:127. doi: 10.1186/s40795-023-00788-9 (PMC10634113; doi:10.1186/s40795-023-00788-9)
Supplement: Supplementary file 1 — Supplementary Material 1 [file 40795_2023_788_MOESM1_ESM.pdf]

ANNEX 1 *Clinical Questionnaire for Assessment of nutritional status*

Part I. Demographic Data

1.	Card No	
2.	Age	
3.	GA	☐term ☐Preterm (____________)
4.	Sex	☐Male ☐Female ☐ Other
5.	Address	
6.	Mother Literacy	☐<8G☐<12G ☐>12G ☐ illiterate
7.	Father Literacy	☐<8G☐<12G ☐>12G ☐ illiterate

Part II: Nutritional Assessment

	**Questions**	
1.	Diagnosis at admission	
2.	comorbidity	☐Down syndrome ☐CHD ☐CP<br>☐ Chemotherapy☐other Specify
**Nutritional history**		
3.	Wt. change in the past 2 weeks	☐no change ☐increased ☐ decreased
4.	Adequacy of dietary intake	☐Adequate ☐inadequate-hypocaloric<br>☐inadequate –starvation (i.e., take little of anything)
5.	Current intake Vs usual	☐no change ☐increased ☐ decreased
6.	Duration of change	☐<2weeks ☐>= 2weeks
7.	Gastrointestinal symptoms: such as anorexia, nausea, abdominal pain, vomiting/gastroesophageal reflux, diarrhea, and constipation.	☐no symptom ☐one or more symptoms not daily ☐some or all symptoms daily
8.	Duration of GI symptom	☐<2weeks ☐>= 2weeks
**Anthropometric measurement's**		
9.	Weight(kg)	

10.	Height(length)(cm)	
11.	MUAc	
**Physical examination**		
12.	Loss of Subcutaneous Fat:(Examine the child's face, arms, chest, and buttocks for loss of subcutaneous fat)	☐no loss in most or all areas<br>☐loss in some but not all areas<br>☐sever loss in most or all areas
13.	Muscle wasting: (Examine the child's temple, clavicle, shoulder, scapula, thigh, knee, and calf)	☐no wasting in most or all areas<br>☐wasting in some but not all areas<br>☐sever wasting in most or all areas
14.	Edema (nutrition related)	☐no edema ☐moderate ☐sever
**Laboratory investigation**		
15.	HB	
16.	Total lymphocyte count	
17.	Albumin	
18.	prealbumin	
<u>**Part III. Surgery and post-op outcome**</u>		
19.	Type of operation	
20.	Intraop accidents, (eg. arrest)	☐yes ☐no
21.	Post-operative hospital stays (days)	
22.	The total length of hospital stays	
**Post op infections**		
23.	SSI	☐yes ☐no
24.	Significant SSI (deep incisional, organ, or space)	☐yes ☐no
25.	Septicemia (objectively defined by positive blood culture,	☐yes ☐no
26.	chest infection proved clinically and radiologically and needing AB treatment	☐yes ☐no
27.	UTI	☐yes ☐no
28.	Others (specify	
**Noninfectious complication**		
29.	Anastomotic leak	☐yes ☐no
30.	Anastomotic stricture	☐yes ☐no
31.	GI bleeding	☐yes ☐no
32.	GI obstruction	☐yes ☐no

33.	Persistent air leak	☐yes ☐no
34.	Atelectasis	☐yes ☐no
35.	Wound dehiscence	☐yes ☐no
36.	Pleural effusion	☐yes ☐no
37.	UCF	☐yes ☐no
38.	gastroenteritis	☐yes ☐no
39.	Others (specify)	
**Up on discharge**		
40.	Weight(kg)	
41.	albumin	
42.	mortality	☐yes ☐no
Follow up- any of the above complications present if yes, write the number		
43.	first	☐yes ☐ no
44.	second	☐yes ☐ no
45.	On 30th post op day	☐yes ☐ no
46.	readmission	☐yes ☐ no
